# Genome-wide association analysis for fumonisin content in maize kernels

**DOI:** 10.1186/s12870-019-1759-1

**Published:** 2019-04-27

**Authors:** L. F. Samayoa, A. Cao, R. Santiago, R. A. Malvar, A. Butrón

**Affiliations:** 1 Misión Biológica de Galicia (MBG - CSIC), Box 28, 36080 Pontevedra, Spain; 2 Facultad de Biología, Department Biología Vegetal y Ciencias del Suelo, Universidad de Vigo, As Lagoas Marcosende, 36310 Vigo, Spain; 3 Agrobiología Ambiental, Calidad de Suelos y Plantas (BVE1-UVIGO), Unidad Asociada a la MBG – CSIC, 36143 Pontevedra, Spain; 4 Present address at department of Crop Science, North Carolina State University, Raleigh, NC 27695 USA

**Keywords:** Maize, *Fusarium verticillioides*, Fumonisin, Resistance, Genome-wide association study, Candidate gene

## Abstract

**Background:**

Plant breeding has been proposed as one of the most effective and environmentally safe methods to control fungal infection and to reduce fumonisin accumulation. However, conventional breeding can be hampered by the complex genetic architecture of resistance to fumonisin accumulation and marker-assisted selection is proposed as an efficient alternative. In the current study, GWAS has been performed for the first time for detecting high-resolution QTL for resistance to fumonisin accumulation in maize kernels complementing published GWAS results for Fusarium ear rot.

**Results:**

Thirty-nine SNPs significantly associated with resistance to fumonisin accumulation in maize kernels were found and clustered into 17 QTL. Novel QTLs for fumonisin content would be at bins 3.02, 5.02, 7.05 and 8.07. Genes with annotated functions probably implicated in resistance to pathogens based on previous studies have been highlighted.

**Conclusions:**

Breeding approaches to fix favorable functional variants for genes implicated in maize immune response signaling may be especially useful to reduce kernel contamination with fumonisins without significantly interfering in mycelia development and growth and, consequently, in the beneficial endophytic behavior of *Fusarium verticillioides*.

**Electronic supplementary material:**

The online version of this article (10.1186/s12870-019-1759-1) contains supplementary material, which is available to authorized users.

## Background

Maize kernels can be contaminated with many mycotoxins produced by different fungi species, most species belonging to the genera *Aspergillus, Penicillium* or *Fusarium*. Concern about kernel contamination with fumonisins is world-wide spread because these toxins are biosynthesized by species of the *Gibberella fujikuroi* complex, such as *Fusarium proliferatum* (Matsushima)*, F. subglutinans* (Wollenw. & Reinking) and *F. verticillioides* (Sacc.) Nirenberg, which infect maize kernels all around the world [1]. Fumonisins have proven toxicity on animals and have been classified as possibly carcinogenic to humans by the International Agency for Research on Cancer [2]. The search for strategies to reduce maize kernel contamination with fumonisins became a priority in many places of the world just few years after fumonisins were discovered [3], and plant breeding has been proposed as one of the most effective and environmentally safe methods to control fungal infection and to reduce fumonisin accumulation [4, 5]. However, conventional breeding can be hampered by the complex genetic architecture of resistance to fumonisin accumulation that appears to be controlled by many quantitative trait loci (QTL) of small effect [1]. In an attempt to avoid this problem, authors have tried to find markers linked to genes involved in resistance to Fusarium ear rot (FER) and/or fumonisin contamination to use them in marker-assisted selection programs [6–12]. Many studies were focused on detecting QTL for resistance to FER; QTL were identified in all chromosomes, except in chromosome 9. However, there are only two studies in which QTL for resistance to fumonisin contamination in maize kernels were located along with QTL for FER; authors pointed out that many QTL detected were associated with both disease traits [8, 13]. As, in addition, genotypic correlation coefficients reported between fumonisin accumulation and FER were high, ranging from 0.87 to 0.99, selection for resistance to FER has been proposed as a simpler method to reduce indirectly kernel contamination with fumonisins [14–17]. However, Eller and coauthors [18] performed selection for resistance to FER and concluded that selection for reduced FER could have limited effectiveness to improve resistance to fumonisin accumulation. In view of these results, more QTL studies to detect specific genomic regions involved in resistance to maize contamination with fumonisins are needed.

QTL mapping using linkage mapping in biparental progenies is a powerful tool to uncover genomic regions involved in the inheritance of a particular trait, but the QTL resolution is low. Therefore, as the lack of tight linkage between markers and QTL could compromise the usefulness of marker-assisted selection (MAS), fine mapping of detected QTL is often addressed before conducting MAS. Fine mapping allows breeders to significantly reduce the confidence interval for QTL position and, at the end, to locate the gene or genes behind the QTL; but it is expensive and time-consuming. In this context, genome-wide association study (GWAS) using inbred line panels appears as an effective alternative to this step-by-step approach for detection of genes involved in resistance to maize kernel contamination with fumonisin. GWAS has been extensively used for detecting associations between molecular markers and resistance to FER or to seedling infection [19–26]. Novel maize loci significantly associated with improved resistance to FER were identified, each locus explaining a small proportion of phenotypic variability. As the alleles conferring greater disease resistance were rare and present in higher frequencies in tropical maize, GWAS has been proposed as a useful tool for identifying specific FER resistance allele variants in tropical maize germplasm to introgress them into temperate dent germplasm [19, 20]. In the current study, GWAS has been performed for the first time for detecting high-resolution QTL for resistance to fumonisin accumulation in maize kernels.

Candidate genes for maize resistance to FER have been proposed in transcriptome, proteome, and metabolome studies deployed to study maize response to infection by *Fusarium verticillioides* in genotypes with contrasting levels of resistance to FER [27–35]. Genes with differential transcript accumulation between resistant and susceptible inbreds at control conditions as well as those specifically induced or downregulated in resistant genotypes after inoculation can be considered as valuable resources to uncover maize resistance mechanisms to FER, especially when they are located in genomic regions containing QTLs. In the present study, this complete information has been taken into account in order to propose candidate genes for the high-resolution QTL detected for fumonisin contamination.

## Results

Genetic heritability for fumonisin content in the kernels (0.42 ± 0.08), estimated on an entry mean basis, was low but significantly different from zero. Genotype x environment interaction was also highly important for this trait (Table 1), but the phenotypic mean across environments would finely correspond to genotype performance because genotype x environment significant effects have been rather attributed to heterogeneity of genotypic variances than to the lack of correlation of genotype performance in different environments [14, 36]. Dispersion of data was higher in 2011 than in 2010 (Additional file 1: Figure S1), but Spearman correlation coefficients between the averaged fumonisin contents and those determined in 2010 and 2011 experiments were 0.834 and 0.830, respectively. BLUE values of inbreds CML158Q, Pa875, CML218, CML228, Mo18W, GT112 and HP301 (belonging to different germplasm groups [37]) were in both years below 10.

**Table 1:** Analysis of variance of a panel of 256 maize inbred lines for fumonisin content in the kernels evaluated in a two-year experiment

Covariance parameters	Estimate	Standard error	*Z* value	*p*-value
Year (Y)	0			
Replication(Y)	179.15	225.60	0.79	0.2136
Block(R*Y)	523.10	365.94	1.43	0.0764
Y*Inbred	2769.75	891.88	3.11	0.0009
Residual	11,189	823.84	13.58	<.0001
Fixed effect	Numerator DF	Denominator DF	*F* value	*p*-value
Inbred	256	235	1.72	< 0.0001

The phenotypic correlation between fumonisin content and FER was not significant (0.40 ± 0.32), meanwhile the genotypic correlation between both traits was higher and significant (0.88 ± 0.11). However, no co-localizations of QTLs for fumonisin content and FER were observed (Data not shown). Phenotypic (− 0.18 ± 0.05) and genotypic (− 0.41 ± 0.11) correlation coefficients between fumonisin content and days to silking were negative and significant.

**Fig. 1:**
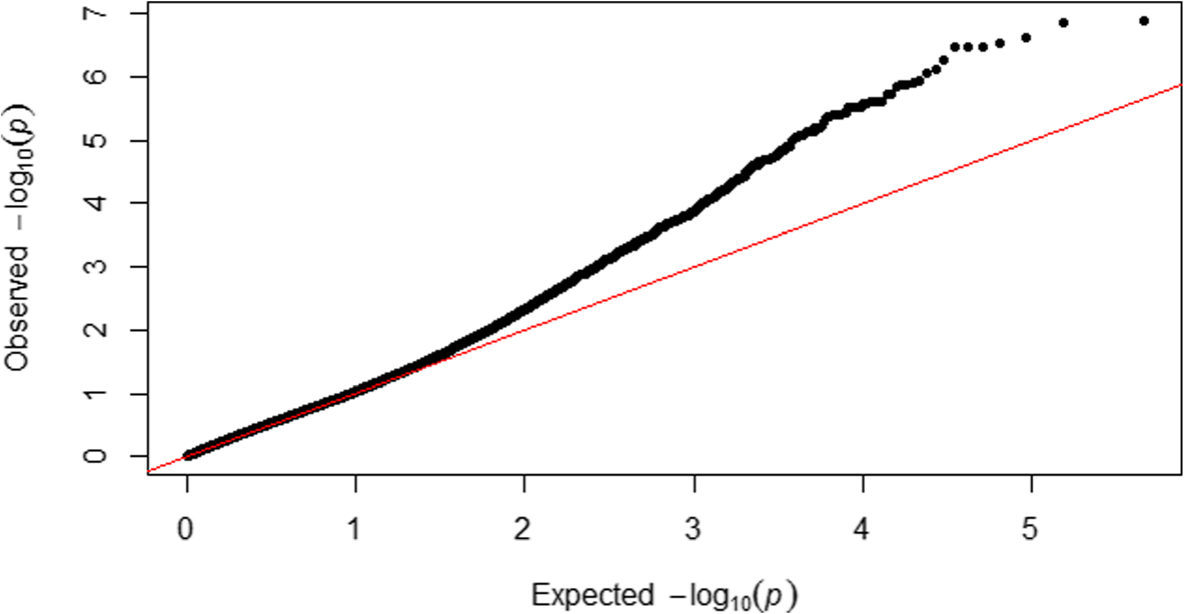
Quantile–quantile plots of a mixed linear model for kernel contamination with fumonisins in a panel of maize inbred lines

**Fig. 2:**
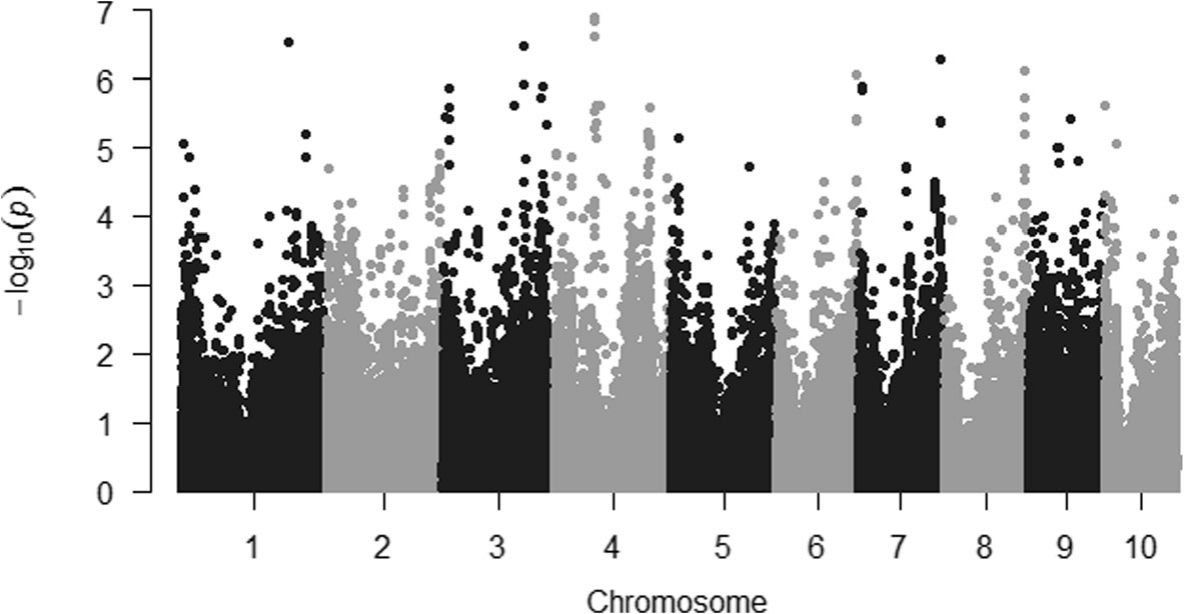
Manhattan plot of a mixed linear model for kernel contamination with fumonisins in a panel of maize inbred lines

**Fig. 3:**
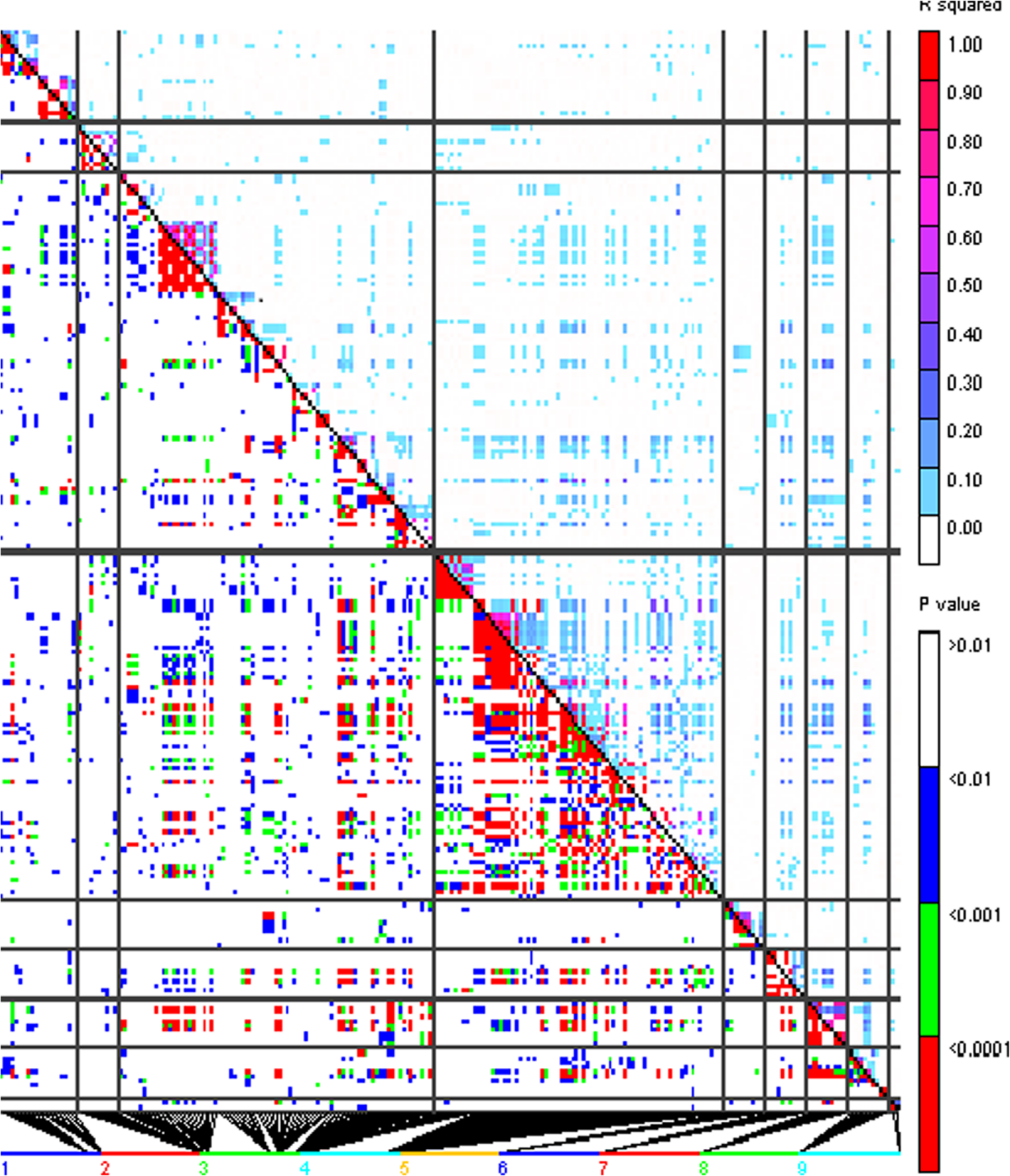
Linkage disequilibrium between SNPs significantly associated with fumonisin content

The 256 inbreds were clustered into 11 groups using the optimum compression option in TASSEL, and the background genetic effects, modeled by the kinship matrix, accounted for the 29% of phenotypic variation for fumonisin content. The “goodness of fit” of the MLM used is shown in the Fig. 1; the outliers, as expected, were situated on the upper part of the Q-Q plot and were scattered across all chromosomes (Figs. 1 and 2). However, only thirty-nine of those outliers surpassed the RMIP threshold of 0.5 and could be considered as reliably associated with fumonisin accumulation in the kernels (Table 2). Significant SNPs were grouped into a unique QTL when they were located in a genomic region in linkage disequilibrium (r^2^ > 0.4), resulting in 17 QTLs for fumonisin accumulation (Table 2). Significant SNPs for resistance to fumonisin accumulation in maize kernels were found in bins 1.07, 1.09, 2.08, 3.02, 3.04, 3.05, 3.06, 3.08, 3.09, 4.02, 4.05, 5.02, 6.07, 7.05, 8.07, 9.03. In general, no LD (r^2^ > 0.4) was found among SNPs associated with different QTLs, except between SNPs in QTLs at chromosomes 3 and 4 (Fig. 3).

**Table 2:** Summary of Genome-wide association study (GWAS) for kernel resistance to fumonisin contamination using a maize inbred panel evaluated under inoculation with *Fusarium verticillioides* in two years

QTL	Bin^1^	Allele^+^	R^2^	QTL SI	SNP position^2^	*p*-value	RMIP	containing-SNP gene_v2^3^	containing-SNP gene_v4
1	1.07	0.95	0.11	220,709,603–222,205,493	220,941,497	9.04*10^−9^	0.80	GRMZM2G078401	Zm00001d032372
2	1.09	0.93	0.08	256,662,974–256,798,243	256,669,820	7.85*10^−7^	0.56	GRMZM2G149028	Zm00001d033386
					256,692,784	2.56*10^−7^	0.63	GRMZM2G100448	Zm00001d033388
					256,692,818	2.54*10^−7^	0.63		
					256,693,243	2.56*10^−7^	0.63		
3	2.08	0.93	0.07	213,583,622–213,815,822	213,588,927	3.84*10^−7^	0.51	GRMZM2G422576	Zm00001d007029
					213,588,940	3.72*10^−7^	0.51		
4	3.02	0.94	0.09	6,970,377–7,206,031	7,119,636	1.25*10^− 8^	0.58	GRMZM2G104176	Zm00001d039513
5	3.04	0.91	0.10	15,040,634–15,127,310	15,056,252	1.85*10^−7^	0.70	GRMZM2G165044	Zm00001d039769
					15,057,326	5.40*10^−9^	0.74		
					15,057,331	2.59*10^−8^	0.64		
					15,057,578	2.25*10^−8^	0.73		
6	3.05	0.93	0.09	147,969,891–148,289,200	147,971,443	4.11*10^−9^	0.69	GRMZM2G701801	Zm00001d042061
7	3.06	0.94	0.11	169,052,376–169,117,125	169,073,710	1.04*10^−10^	0.60	GRMZM2G026855	Zm00001d042555
					169,073,715	1.04*10^−10^	0.60		
					169,073,720	1.05*10^−10^	0.60		
					169,078,446	4.77*10^−9^	0.56		
8	3.06	0.93	0.08	173,006,714–173,381,106	173,283,279	5.89*10^−7^	0.53	GRMZM2G060255	Zm00001d042658
9	3.08	0.91	0.10	206,261,823–207,246,656	206,605,245	1.22*10^−6^	0.56	GRMZM2G169654	Zm00001d043782
						4.68*10^−8^	0.62	GRMZM2G028467	Zm00001d043801
10	3.09	0.89	0.09	217,324,678–217,572,395	217,558,124	2.46*10^−8^	0.53	**GRMZM2G042421**	**Zm00001d044173**
11	4.02	0.93	0.08	5,405,928–5,466,378	5,406,694	2.03*10^−7^	0.57	GRMZM2G154414	Zm00001d048837
					5,410,388	2.09*10^−7^	0.55		
12	4.05	0.87	0.12	centromere	82,892,426	3.60*10^−8^	0.58		
					82,892,436	3.60*10^−8^	0.58		
					82,892,557	3.60*10^−8^	0.58	GRMZM2G111117	Zm00001d050400
					83,027,805	3.60*10^−8^	0.58	GRMZM2G178169	Zm00001d050401
					83,033,318	3.60*10^−8^	0.58		
					83,572,578	2.10*10^−8^	0.74	AC198937.4_FG005	Zm00001d050410
					83,738,156	2.11*10^−8^	0.77		
					84,453,345	3.44*10^−8^	0.73	GRMZM2G123362	Zm00001d050434
					88,069,675	2.54*10^−8^	0.56		
					88,098,841	2.50*10^−8^	0.52		
					96,545,683	1.17*10^−7^	0.54	GRMZM2G140095	Zm00001d050575
13	5.02	0.95	0.08	14,815,481–14,839,860	14,839,828	6.99*10^−9^	0.58	**GRMZM2G066449**	**Zm00001d013611**
14	6.07	0.90	0.10	164,365,931–164,378,580	164,369,763	3.76*10^−9^	0.61	GRMZM2G038183	Zm00001d038998
15	7.05	0.93	0.10	171,058,866–171,182,450	171,182,417	2.89*10^−9^	0.64	GRMZM2G009021	Zm00001d022400
16	8.07	0.85	0.10	167,189,708–167,286,546	167,284,361	3.19*10^−9^	0.54	**GRMZM2G177324**	**Zm00001d012329**
17	9.03	0.91	0.09	centromere	87,360,835	1.88*10^−8^	0.50	GRMZM2G159641	Zm00001d046455

^1^Bin in which QTLs are located; Allele^+^ stands up for the frequency of favorable allele; R^2^ for the proportion of the phenotypic variance explained by the QTL; QTL SI for the supporting interval of the QTL on the RefGenB73_v2, region in which appreciable linkage disequilibrium is observed between SNPs (r^2^ > 0.4), SI could not be visualized using LD plots from TASSEL when SNP were localized in centromeric regions where LD is extremely high

^2^SNP position for the position in bp of the significant SNP on the RefGenB73_v2; *p*-value for the association between polymorphic variation at the SNP and phenotypic variation for fumonisin content; RMIP for resample model inclusion probability

^3^Filtered genes in the RefGenB73_v2 and RefGenB73_v4 versions of the B73 sequence that contain the significant SNP (or the closest filtered gene in bold)

The supporting intervals for the QTL ranged from thousands to millions of bp and were positioned in the B73 genome v2 (RefGen_v2) (ftp://ftp.ensemblgenomes.org/pub/plants/release-7/fasta/zea_mays/dna/) as well as in the B73 genome v4 (RefGen_v4) [38] (Table 2 and Additional file 2: Table S1). All genes located within the supporting interval (based on RefGen_v4) of each QTL were considered as candidate genes for that QTL (Additional file 2: Table S1), and genes with annotated functions probably implicated in resistance to pathogens based on previous studies will be discussed. No candidate genes, except the SNP-containing genes, are proposed for QTL located in genomic regions where linkage disequilibrium is high and confidence interval spans more than 2 Mbp, such as those in bins 4.05 and 9.03.

## Discussion

Differences among inbreds for fumonisin content in the kernels were significant and the genetic heritability for fumonisin content in the kernels was low but significantly different from zero showing that there is additive genetic variability among inbreds for resistance to fumonisin accumulation. The heritability for fumonisin content was similar to those reported by Hung and Holland [39], but smaller than those observed in genetically narrower populations [14–16]. Low heritability for fumonisin contamination stresses the importance of implementing marker-assisted selection methods based on stable QTLs in order to increase maize resistance to kernel contamination. In this scenario, marker-assisted selection would be even more efficient than arduous and expensive selection programs based on the phenotype.

The lack of significant phenotypic correlation between fumonisin content and FER could be due to low pathogenicity of the isolate or/and climatic conditions that would not be favorable for disease spread since, in the same experiments, reported FER values for the same inbreds were moderate [19], while those conditions would be more favorable for fumonisin accumulation because the average mean for kernel contamination was 58.4 ppm [one third of inbreds presented mean values above 50 ppm, meanwhile approximately 10% of inbreds presented values below 10 ppm]. Then, conducive conditions for fumonisin accumulation but not for disease development could account for the lack of phenotypic correlation between both traits, contrarily to reported results [1], and no detection of QTL for FER [19] using the same experimental trials.

In previous studies, positive correlation coefficients between days to silking and fumonisin accumulation were found [13, 14]; meanwhile, in the current study, the genotypic correlation coefficient between fumonisin content and days to silking was negative. However, co-localization of QTLs for fumonisin content (Table 2) and days to silking (data not shown) occurred in the interval 5,405,928-5,466,378 of chromosome 4 and alleles for increased fumonisin content and days to silking appeared to be linked in coupling phase. We hypothesize that population structure could be responsible for the significant and positive genotypic correlation coefficient observed between both traits in the current study because tropical maize inbreds are later and show higher frequencies for resistance alleles to FER [20]. Therefore, after removing random genetic variation (variation explained by additive relationship matrix), linked genetic variants for increased accumulation of fumonisin and delayed maturity can be found.

The 39 SNPs significantly associated with fumonisin accumulation in the maize kernels were grouped in 17 high-resolution QTLs and, at least, four of them would be behind novel QTL not reported in previous studies [8, 13]. These novel QTLs for fumonisin content would be at bins 3.02, 5.02, 7.05 and 8.07. Genomic regions significantly associated with FER in previous GWAS did not overlap, in general with QTL supporting intervals for kernel contamination with fumonisins, excepting particular regions in bins 3.08, 4.05, 7.05, and 9.03 [19–25]. Therefore, These QTL could be especially useful to reduce kernel contamination with fumonisins without significantly interfering in mycelia development and growth and, consequently, in the known beneficial endophytic behavior of *Fusarium verticillioides. Fusarium verticillioides* has already been proved as contributor to host fitness through growth promotion and induction of defense-associated changes such as lignin deposition in the cell wall at seedling stage and growth increased in mature plants [40–43]. However, due to the polygenic nature of genetics for maize fumonisin contamination, breeding should rather be based on genomic selection (GS) models than on marker-assisted approaches focused on fixing exclusively favorable genetic variants for the QTL detected. However, precisely mapped QTL could improve genomic prediction accuracy using stepwise linear regression mixed model to unify GWAS and GS in a single statistical model [44].

### Candidate genes

QTL supporting interval comprises the QTL-surrounding region in LD (*r*^*2*^ > 0.4). All genes contained in the supporting interval were considered as candidate genes and identified and characterized by the use of the MaizeGDB genome browser. However, discussion will be mainly focused on genes with annotated functions probably implicated in resistance to pathogens.

Toxin biosynthesis seems to be coupled to colonization of the host and some *Fusarium verticillioides* genes with important roles in both processes have been characterized [40, 45, 46]. For example, the gene FUG1 plays a role in mitigating stresses associated with the host environment, being a critical component of the genetic regulatory network underlying maize kernel pathogenesis and fumonisin biosynthesis. Accordingly, it is expected that some host genes involved in defense against fungal disease would be also implicated in toxin modulation.

Plants have an innate immunity system to defend themselves against pathogens [47]. Pattern triggered immunity (PTI) or basal defense response is mediated by plant pattern recognition receptors (PRRs) that recognize pathogen-associated molecular patterns (PAMPs), but plant pathogens can suppress this basal defense response by effectors which contribute to pathogen virulence. However, a secondary immune response, effector-triggered immunity (ETI) mediated by resistance proteins (RPs) that recognize effector-induced perturbations of host targets, allows plants to stop pathogen growth. In addition, during induction of local immune responses, systemic acquired resistance (SAR) can become activated. PTI seems to play a primary role in the resistance of maize to *Fusarium verticillioides,* and maize resistance would be achieved somehow through PTI-induced acquired systemic immunity where ABA, SA, and JA hormone signaling pathways can be involved [33]. Therefore, genes directly implicated in the immune plant response deserve special attention as preferred candidate genes for the significant associations found.

Zm00001d042659 (at ≈ 175 Mbp in chromosome 3 of the RefGenB73_v4) has been annotated as a *protein SRC2-like protein* gene and, consequently, could be implicated in recognition of PAMPs because, in pepper, a SRC2 protein acts as a required interacting partner of a fungal elicitor of the immune response [48]. L-type lectin-domain containing receptor kinases have been proposed as plant sensors of pathogen invasion and, consequently, the gene Zm00001d043781, annotated as an *L-type lectin-domain containing receptor kinase IV.1* gene, could be a good candidate gene for the QTL at 3.08 [49].

The largest class of resistance proteins involved in ETI response consists of nucleotide-binding-leucine rich repeat (NB-LRR) proteins. In *Arabidopsis*, the gene *LOV1* encodes a typical NB-LRR but this protein is unique because it confers sensitivity to the fungal toxin victorin and susceptibility to the fungus *Cochliobolus victoriae*. In the current study, a putative *inactive disease susceptibility protein LOV1* (Zm00001d032376) gene is located within the confidence interval of QTL at 1.07 and is proposed as probable candidate gene for that QTL.

Similarly, genes involved in plant immune response signaling could contribute to plant resistance. Salicylic acid is a defense hormone required for both local and systemic acquired resistance (SAR) in plants. Salycilic acid is synthesized from chorismate, the end product of the shikimate pathway, although the complete biosynthetic route has yet to be established. Then, genes involved in chorismate biosynthesis and in the response to pathogen effector proteins, such as *phospho-2-dehydro-3-deoxyheptonate aldolase* genes, are good candidate genes for the QTL detected [50, 51]. Gene Zm00001d013611 has been proposed as a *phospho-2-dehydro-3-deoxyheptonate aldolase 2, chloroplastic-like* gene and could be behind the QTL at 5.02. Besides structural genes of the chorismate pathway, genes with proven regulatory role can be highlighted as candidate genes. Zm00001d032368 which codifies for Protein SAR DEFICIENT 1 (SARD1) is a good candidate for the QTL found in 1.07 because SARD1 has been reported as a positive regulator required for salicylic acid accumulation [52]. The gene Zm00001d033389 is proposed as the preferred candidate gene for QTL at 1.09 (contained in the confidence interval of the QTL that spans from 261,226,685 to 261,380,422 in RefGen_v4) because codifies for a VQ motif family protein. Members of the VQ family play either positive or negative roles in SA- and/or JA-mediated plant immune responses [53].

As auxin can interfere with plant defense circuitry through antagonism with SA signaling [54], another set of interesting genes for future validations comprised genes with proven or probable functions in auxin signaling [Zm00001d039513 (*Aux/IAA-transcription factor 7* at bin 3.02), Zm00001d044172 (*srph1 - SGT1 disease resistance homolog1*at bin 3.09), and Zm00001d022400 (*F-box protein SKIP5* gene at bin 7.05)] [55] and auxin signal transduction [Zm00001d048841 (probable *patatin-like phospholipase* gene at bin 4.02)] [56]. The possible effect of genes modulating auxin signaling and transport on maize seedling resistance to Gibberella stalk rot caused by *Fusarium graminearum* has already been shown [57]. In general, modulation of plant disease resistance by auxin and/or its signaling pathway has been proposed based on results from many pathogen-host interactions [54]. Finally, it has also been shown that canonical cell cycle regulators such as cyclin-dependent kinase inhibitors form part of signaling pathway directly involved in ETI and could also contribute to basal resistance [58]. Therefore, Zm00001d048837 and Zm00001d013610 annotated as likely cyclin-dependent kinase inhibitors could be stressed as candidate genes for QTL at 4.02 and 5.02, respectively, and deserve especial attention.

In addition to salicylic acid, plant lipid metabolites are important signal molecules in local and systemic defense against pathogens [59]. More specifically, fungal and plant oxylipins (including the well-known jasmonic acid), produced via the oxidation of polyunsaturated fatty acids, have a primordial role as signals in plant–pathogen ecosystems [60]. Fungal oxylipins attempt to reprogram PTI and, in turn, the host counteracts by producing its own oxylipins to impede pathogen infection: However, fungal oxylipins can also induce Effector Triggered Susceptibility (ETS) by activating genes of the host oxylipin pathway, such as *ZmLOX3,* that suppress defense-related branches of the maize oxylipin pathway and favor *Fusarium verticillioides* virulence and fumonisin accumulation [60, 61]. Sphingolipids, also play an important role in the regulation of the delicate arm race between the microbe and the host in mammals. A similar involvement of sphingolipids in immune plant response signaling could be hypothesized based on scarce studies that identify genes implicated in sphingolipid metabolism as important factors in resistance to fungal infection [62, 63]. Under field conditions, it has been stablished that oxilipin and sphingolipid metabolism in maize kernels interferes with *Fusarium verticillioides* growth and fumonisin production; early activation of plant lipoxygenase genes and genes for jasmonic acid biosynthesis appear important factors for conferring resistance [35, 64, 65]. Therefore, Zm00001d039768 (Acyl-coenzyme A oxidase 4 peroxisomal gene) is proposed for the QTL at 3.04 which contains significant SNPs S3_15,056,252, S3_15,057,326, S3_15,057,331, and S3_15,057,578; and Zm00001d044175 (Neutral/alkaline non-lysosomal ceramidase gene) is proposed for the QTL at 3.09.

Finally, another lipid component of the plant, the cuticle, could also play an important role in plant defense against attack by fungi. The plant cuticle is a protective sheathing produced by epidermal cells of aerial plant organs that provides the first barrier that fungi must overcome in order to get into the plant tissue. However, the cuticle also provides chemical and physical cues that are necessary for the development of essential infection structures for many fungal pathogens and perception of cuticle alterations by fungi could be essential for promoting plant defenses [66]. In rice, an abnormal cuticle formation may affect the signaling of plant defense against the hemibiotrophic fungus, *Magnaporthe oryzae* [67]. Therefore, the gene *myb28* (Zm00001d050400) which is orthologous to the *Atmyb16* gene that participates in the regulation of cuticle biosynthesis in *Arabidopsis* [68] could be a good candidate for the QTL at 4.05.

There are numerous pathogenesis-related changes that follow PAMP perception, such as rapid in fluxes of cytosolic Ca^+2^and production/accumulation of reactive oxygen species (ROS). Genes involved in protection of plant tissues against oxidative damage and ROS detoxification could be important in maize defense against *Fusarium verticillioides*; the constitutive higher antioxidant content in resistant genotypes seeming crucial in maize kernels in preparation of pathogen attack [34]. Therefore, genes involved in ROS production and ROS-scavenging and ROS-detoxification could be also good candidates: Zm00001d042061 (a probable NADPH: quinone oxodoreductase gene) was suggested as candidate gene for the QTL at 3.05; Zm00001d042555 (a putative alcohol dehydrogenase gene) for the QTL at ≈ 171 Mbp in chromosome 3 of the RefGenB73_v4 (bin 3.06); Zm00001d043787, Zm00001d043789, and Zm00001d043795 (glutathione transferase genes) and Zm00001d043782 [*ZmRav1*, that might improve stress tolerance through the regulation of the expression of genes involved in ROS scavenging [69]] for the QTL at 3.08; and the gene Zm00001d046455 (a gene codifying for a protein with predicted oxidoreductase and transferase activities) for the QTL at 9.03.

Lanubille and coauthors observed that the response of a resistant genotype to kernel infection by *Fusarium verticillioides* was characterized by a constitutive expression, and by a prompt and enhanced induction of some key genes [30]. Therefore, candidate genes in the current GWAS, that were differentially transcribed at control conditions in resistant and susceptible genotypes as well as those specifically modified by *Fusarium verticillioides* infection in the resistant genotype in the work by Lanubile et al. [30] (Zm00001d007025, Zm00001d007032 and Zm00001d043798), would deserve especial attention. The functions of genes Zm00001d007025 and Zm00001d007032 (named previously GRMZM2G422537 and GRMZM2G035356, respectively) are unknown; while Zm00001d043798 (named before GRMZM2G448710), a *Leaf rust 10 disease-resistance locus receptor-like protein kinase gene*, could be involved in basal defense against fungi [70].

## Conclusions

Complexity of genetics of maize resistance to kernel contamination with fumonisins has been confirmed because genotype x environment interaction had an important contribution to phenotypic variation and many genes with small effects would contribute to genetic variation. Thirty-nine SNPs significantly associated with resistance to fumonisin accumulation in maize kernels were found and clustered into 17 QTL. Novel QTLs for fumonisin content would be at bins 3.02, 5.02, 7.05 and 8.07. The high resolution of QTLs found using GWAS allows us to propose candidate genes for these QTLs; many candidates being implicated in maize immune response signaling. Functional variation for those genes may be especially useful to reduce kernel contamination with fumonisins without significantly interfering in mycelia development and growth and, consequently, in the beneficial endophytic behavior of *Fusarium verticillioides*. Validations of the contributions of these candidate genes to resistance to fuminisin accumulation in maize kernels will be the focus of future works.

## Methods

### Plant material and field experiments

A subset of 270 inbred lines from a maize diversity panel (composed of 302 inbred lines) that represents much of the diversity available in public breeding sector around the world [71] was evaluated in 2010 and 2011 under inoculation with *Fusarium verticillioides*. Seeds were provided by the North Central Regional Plant Introduction Station (NCRPIS) in Ames, Iowa, and NCRPIS accession names are shown in Additional file 3 Table S2.

Evaluations were done at Pontevedra (42°24′ N, 8°38′ W, and 20 m above sea level), Spain, using an 18 × 15 α-lattice design with two replications. Trials were hand-planted and each experimental plot consisted of one row spaced 0.8 m apart from the other row with 29 two-kernel hills spaced 0.18 m apart. Plots were overplanted and thinned, obtaining a final density of ~ 70,000 plant ha^− 1^. In each row, between seven and 14 days after silking date, five primary ears were inoculated with two milliliters of a spore suspension of a local toxigenic isolate of *Fusarium verticillioides* using a tested kernel inoculation protocol [72]. The spore suspension contained 10^6^ spores per milliliter and was injected into the center of the ear using a four-needle vaccinator. Inoculated ears from each row were collected 2 months after inoculation, dried at 35 °C for 1 week, and shelled. From each plot, a representative kernel sample of approximately 200 g was ground and stored at 4 °C until performing chemical analyses. Kernels were ground through a 0.75 mm screen in a Pulverisette 14 rotor mill (Fritsch GmbH, Oberstein, Germany).

Ground samples were sent to the Food Technology Department of the University of Lleida, Spain, for determination of total fumonisin (fumonisins B_1_, B_2_, and B_3_) content using a commercial ELISA kit (R-Biopharm Rhône Ltd., Glasgow, Scotland, UK). This kit is a competitive enzyme immunoassay for quantification of fumonisin residues in maize. The recovery rate of the test was approximately 60% with a mean coefficient of variation of approximately 8%; specifities for B_1_, B_2_, and B_3_ were 100%, around 40%, and almost 100%, respectively, and the detection limit was 0.025 ppm (mg kg^− 1^). Extraction and preparation of samples, as well as test performance, were carried out as described in the commercial kits.

### Genotypic data

We used the genotypes of 256 inbred lines with phenotypic data in both years for a set of approximately 990,000 SNP markers (AllZeaGBSv2.7) derived from a genotyping-by-sequencing (GBS) strategy (Elshire et al. 2011) and uplifted to AGPv3 (Glaubitz et al. 2014) [73]. SNPs in chromosome 0, as well as monomeric and multiallelic SNPs and insertion/deletion polymorphisms (INDELs) were excluded. Then, data set was first filtered to exclude SNPs with more than 20% missing genotype data, and minor allele frequency (MAF) less than 5%. After performing imputation with Beagle v4.0 (Browning and Browning 2016), a second filtering (missing > 20% and MAF < 5%) was done after setting heterozygous genotypes as missing in the analysis. A total of 226,446 filtered SNPs distributed across the maize genome were used for GWAS analysis. After performing a linkage disequilibrium-based pruning in software Plink v1.9 a subset of ~ 99 k SNPs was obtained and used to perform a kinship matrix (K) in Tassel 5.

### Statistical analyses

Heritabilities (${\widehat{h}}^2$) across environments were estimated for fumonisin contamination on a family-mean basis as described by Holland et al. [74]. The genetic and phenotypic correlations between fumonisin content and other data previously published [75], days to silking and FER, were computed following Holland [76]. Best linear unbiased estimator (BLUE) was estimated for each inbred line using the SAS mixed model procedure (PROC MIXED) and considering inbred line as fixed effect and replication within year, block within replication*year and year as random effects. Line BLUEs were used to perform GWAS.

Genome-wide association analysis based on mixed linear model (MLM) was performed in Tassel V5.2.25 [77]. The MLM used by Tassel was

$$ \mathrm{y}=\mathrm{X}\upbeta +\mathrm{Zu}+\mathrm{e} $$

where **y** is the vector of phenotypes (BLUEs), **β** is a vector of fixed effects, including the SNP marker tested, **u** is a vector of random additive effects (inbred lines), **X** and **Z** represents matrices, and e is a vector of random residuals. The variance of random line effects was modeled as Var(u) = **K** ${\sigma}_a^2$, where **K** is the *n* × *n* matrix of pairwise kinship coefficient and ${\sigma}_a^2$ is the estimated additive genetic variance [78]. Restricted maximum likelihood estimates of variance components were obtained by using the optimum compression level (compressed MLM) and population parameters previously determined options (P3D) in Tassel [79].

To identify SNPs with the most robust associations with traits, a subsampling or subagging procedure was employed in GWAS analysis [80, 81]. Each of 100 subsampled datasets generated using the R software [82] comprised a random sample of 80% of inbred lines from the diversity population. Only SNP markers determined as significant at *p* < 1 × 10^− 4^ and subsequently detected in ≥50 subsamples, i.e. resample model inclusion probability (RMIP) threshold of 0.50, were considered as significantly associated with the trait under study. Analysis of linkage disequilibrium (LD) among SNPs significantly associated with fumonisin content was performed in Tassel.

### Candidate gene selection

We also examined the LD in the genomic region around each significant SNP to stablish a supporting interval for the significant association. That supporting interval would comprise the surrounding region in LD (*r*^*2*^ > 0.4). All genes contained in the supporting interval were considered as candidate genes and identified and characterized by the use of the MaizeGDB genome browser [83]. Although SNP positions were referenced to the maize B73 RefGen_v2, the genes flanking the region in LD were positioned in the maize B73 RefGen_v4 to perform the search for candidate genes in the latest version of the B73 sequence.

## Additional files

Additional file 1: **Figure S1.** Data distribution for fumonisin content in 2010 (left) and 2011 (right). (PNG 22 kb)

Additional file 2: **Table S1.** Candidate Genes for each QTL. (XLSX 15 kb)

Additional file 3: **Table S2.** Names of the panel inbreds along with their accession identifications at the North Central Regional Plant Introduction Station (NCRPIS). (XLS 40 kb)

## Abbreviations

FER: Fusarium Ear Rot
GS: Genomic selection
GWAS: Genome-Wide Association Study
LD: Linkage Disequilibrium
MAS: Marker-Assisted Selection
QTLs: Quantitative Trait Loci
SNP: Single Nucleotide Polymorphism
