# Supplementary figures and images for: Genome-wide association analysis for fumonisin content in maize kernels
Source: BMC Plant Biol. 2019 Apr 27;19:166. doi: 10.1186/s12870-019-1759-1 (PMC6486958; doi:10.1186/s12870-019-1759-1)

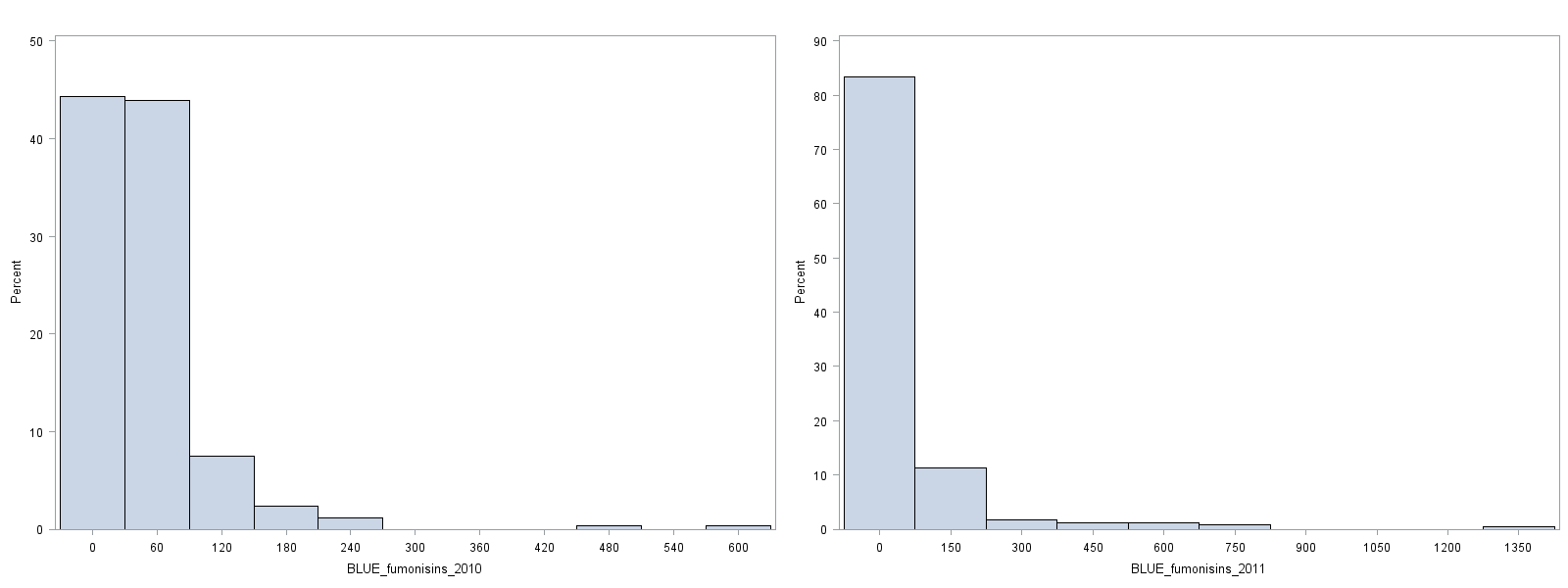

Supplement: Supplementary file 1 — Figure S1. Data distribution for fumonisin content in 2010 (left) and 2011 (right). (PNG 22 kb) [file 12870_2019_1759_MOESM1_ESM.png]
